# Searching for G-Quadruplex-Binding Proteins in Plants: New Insight into Possible G-Quadruplex Regulation

**DOI:** 10.3390/biotech10040020

**Published:** 2021-09-22

**Authors:** Adriana Volná, Martin Bartas, Jakub Nezval, Vladimír Špunda, Petr Pečinka, Jiří Červeň

**Affiliations:** 1 Department of Physics, Faculty of Science, University of Ostrava, 710 00 Ostrava, Czech Republic; adriana.volna@osu.cz (A.V.); jakub.nezval@osu.cz (J.N.); vladimir.spunda@osu.cz (V.Š.); 2 Department of Biology and Ecology, Faculty of Science, University of Ostrava, 710 00 Ostrava, Czech Republic; martin.bartas@osu.cz (M.B.); petr.pecinka@osu.cz (P.P.); 3 Global Change Research Institute, Czech Academy of Sciences, Bělidla 4a, 603 00 Brno, Czech Republic

**Keywords:** G-quadruplex-binding proteins, RGG motif, NIQI, G-quadruplex folding, G-quadruplex resolving, regulation of gene expression

## Abstract

G-quadruplexes are four-stranded nucleic acid structures occurring in the genomes of all living organisms and viruses. It is increasingly evident that these structures play important molecular roles; generally, by modulating gene expression and overall genome integrity. For a long period, G-quadruplexes have been studied specifically in the context of human promoters, telomeres, and associated diseases (cancers, neurological disorders). Several of the proteins for binding G-quadruplexes are known, providing promising targets for influencing G-quadruplex-related processes in organisms. Nonetheless, in plants, only a small number of G-quadruplex binding proteins have been described to date. Thus, we aimed to bioinformatically inspect the available protein sequences to find the best protein candidates with the potential to bind G-quadruplexes. Two similar glycine and arginine-rich G-quadruplex-binding motifs were described in humans. The first is the so-called “RGG motif”-RRGDGRRRGGGGRGQGGRGRGGGFKG, and the second (which has been recently described) is known as the “NIQI motif”-RGRGRGRGGGSGGSGGRGRG. Using this general knowledge, we searched for plant proteins containing the above mentioned motifs, using two independent approaches (BLASTp and FIMO scanning), and revealed many proteins containing the G4-binding motif(s). Our research also revealed the core proteins involved in G4 folding and resolving in green plants, algae, and the key plant model organism, *Arabidopsis thaliana*. The discovered protein candidates were annotated using STRINGdb and sorted by their molecular and physiological roles in simple schemes. Our results point to the significant role of G4-binding proteins in the regulation of gene expression in plants.

## 1. Introduction

**Figure 1:**
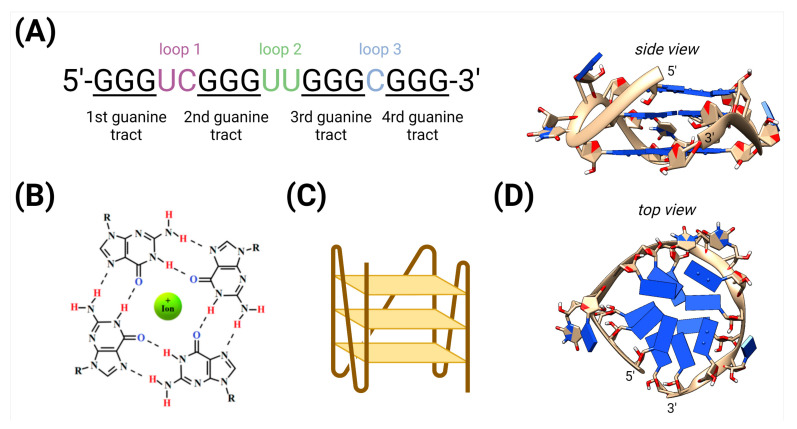
The G4 forming sequence. (**A**) The G4 forming sequence consists of four consecutive guanine tracts (underlined), separated by other nucleotides (that later form loops 1–3). (**B**) Guanine tetrad stabilized by Hoogsteen base pairing and positively charged central ion. (**C**) Schematic drawing of the parallel G4 structure consisting of three stacked guanine tetrads, created with BioRender. (**D**) A 3D model of the intramolecular parallel RNA G4 formed from the sequence depicted here, modeled using the 3D-NuS web server [6].

G-quadruplexes (G4s) are secondary structures of nucleic acids that can arise in guanine-rich DNA or RNA regions [1]. Each G4 is formed by its basic units, termed guanine tetrads (see Figure 1). A single guanine tetrad consists of four guanine nucleotides interconnected by Hoogsteen base pairing. G4s are further stabilized by the positively charged monovalent ions that are localized in their central cavity [2]. Although there are common properties in G4s, there is also great structural diversity in their composition, including in the number of planes, loop lengths, the orientation of tracts, etc. [3,4,5].

In humans, G4-forming sequences have been found in the genes that are important for key cellular processes [1], and it appears that they play an important role in the development of cancer [7] and neurodegenerative diseases [8,9]. Additionally, G4s may play a role in viral lifecycles [10], including that of novel SARS-CoV-2 [11]. It is clear that the formation of G4 structures itself is not sufficient to support all of its functions, and the binding of specific proteins (including DNA/RNA-binding proteins) often works as a trigger and/or modulator of their effects. Currently, nearly 100 G4-binding proteins are known to be present in humans and other model organisms [12,13].

Although the number of research papers dealing with G4s in humans is exponentially rising, there is still only limited evidence regarding G4s in plants. Recently, genome-wide studies were performed on barley [14] and wheat [15], and the partial knowledge about other agriculturally important plants was reviewed [16]. The G4s were associated with a large range of important molecular processes, such as the regulation of transcription, translation, the response to various types of stresses, and even plant-specific processes (such as flowering and phloem formation) [16]. G4s were also discussed as a possible UV sensor in plants [17], and, lastly, previous research suggested that G4s might be responsible for the formation of the *SHORT ROOT* RNA phase-separation-like phenomenon in vivo [18].

Even less is known about G4-binding proteins in plants. In 2015, Andorf et al. described nucleoside-binding kinase 1 *ZmNDPK1* as the first known G4-binding protein in plants [19]. Cho et al. described the binding of the protein, JULGI, to RNA G4 located in 5’UTR of the *SMXL4* and *SMXL5* genes inhibiting their translation [20]. Additionally, Sjakste et al. analyzed the sequences of DNAs that were tightly bound to the proteins in barley seedlings [21]. They found that the sequences that were bound by these proteins were highly enriched in GC content, compared to the rest of the barley genome, and CD spectroscopy confirmed that JULGI-bound sequences are able to form G4s [20].

Functional and structural studies of G4-binding proteins in plants are absent, and this interesting topic should be studied. Therefore, we aimed to computationally identify the potential G4-binding proteins in plant genomes, using the experimentally validated RGG region of protein FMRP (which is known to interact with G4s [22]), and the so-called Novel Interesting Quadruplex Interaction (NIQI) motif, which is common in many G4-binding proteins in humans [23].

The main aim of this study was to inspect whether plants (*Viridiplantae*, both green plants and algae) dispose of the proteins containing RGG and NIQI motifs with ability to bind G4s and, thus, to make a preselection of suitable candidates for further wet-lab testing. For these purposes, we used two distinct bioinformatic tools: BLASTp and FIMO. Lastly, we identified which molecular and physiological processes our protein candidates are involved in.

## 2. Materials and Methods

### 2.1. Identification of G4-Binding Proteins Using the FIMO Approach

Identification of G4-binding proteins in the model plant organism *Arabidopsis thaliana* was performed using FIMO (https://meme-suite.org/meme/tools/fimo accessed on 26 January 2021) [24], which is part of the MEME Suite [25], with a custom threshold of *p* = 1 × 10^−9^ to minimize false-positive results (default value is 1 × 10^−4^). As the input for motif scanning, we used reference *Arabidopsis thaliana* proteome (40 885 proteins from the RefSeq NCBI database [26]). Motif scanning was carried out for both previously identified G4-binding motifs (NIQI-“RGRGRGRGGGSGGSGGRGRG” and experimentally verified RGG motif from the FMRP protein “RRGDGRRRGGGGRGQGGRGRGGGFKG”). Subsequently, we repeated the whole analysis for all members of the *Viridiplantae* group, comprising both green plants and algae.

### 2.2. Identification of G4-Binding Proteins Using BLASTp Approach

As an alternative method for the comparison of FIMO results, we performed BLASTp (https://blast.ncbi.nlm.nih.gov/Blast.cgi?PAGE=Proteins accessed on 30 July 2021) [27] searching for both G4-binding sequences separately (NIQI and RGG). At first, search was limited to the organism *Arabidopsis thaliana* (non-redundant protein sequences database (nr), default parameters), and then the search was extended to the entire *Viridiplantae* group, comprising both green plants and green algae (nr database).

### 2.3. Functional Interaction Network Analysis Using STRING Approach

Functional interaction networks of proteins containing the RGG [28] or NIQI [23] motif were constructed using the STRING (https://string-db.org/ accessed on 20 February 2021) [29] online tool with the following parameters: disable structure previews in bubbles, hide disconnected nodes in network, show input protein names, k-means clustering with 3 nodes, and other parameters (including interaction score) were set as default.

### 2.4. Process of 3D Structural Modeling and Docking

The 3D models of selected proteins were prepared using RaptorX web server (http://raptorx.uchicago.edu/ accessed on 18 May 2021) [30] for proteins shorter than 1100 aa. For longer proteins (>1100 aa), EsyPred3D was used (https://www.unamur.be/sciences/biologie/urbm/bioinfo/esypred/ accessed on 19 May 2021) [31]. In both cases, the resulting 3D structures were exported in .pdb files and uploaded to the HDOCK server (http://hdock.phys.hust.edu.cn/ accessed on 25 May 2021) [32] to model protein-G4 docking. The representative RNA G4 structure was modeled using the 3D-NuS web server (https://iith.ac.in/3dnus/ accessed on 4 June 2021) [6], and, as an input, the G4-forming sequence from [33] was used. Structures were visualized using the UCSF Chimera standalone tool [34]. Prediction of clashes/contacts was also carried out within this tool, with the following parameters: “VDW overlap” ≥ 0.4 angstroms; “subtractions of 0.4 from overlap for potentially H-bonding pairs”; “Ignoring contacts of pairs 2 or fewer bonds apart”; “Including intra-molecule contacts”.

## 3. Results

### 3.1. Identification of G4-Binding Proteins in Plants Using a FIMO Approach

**Figure 2:**
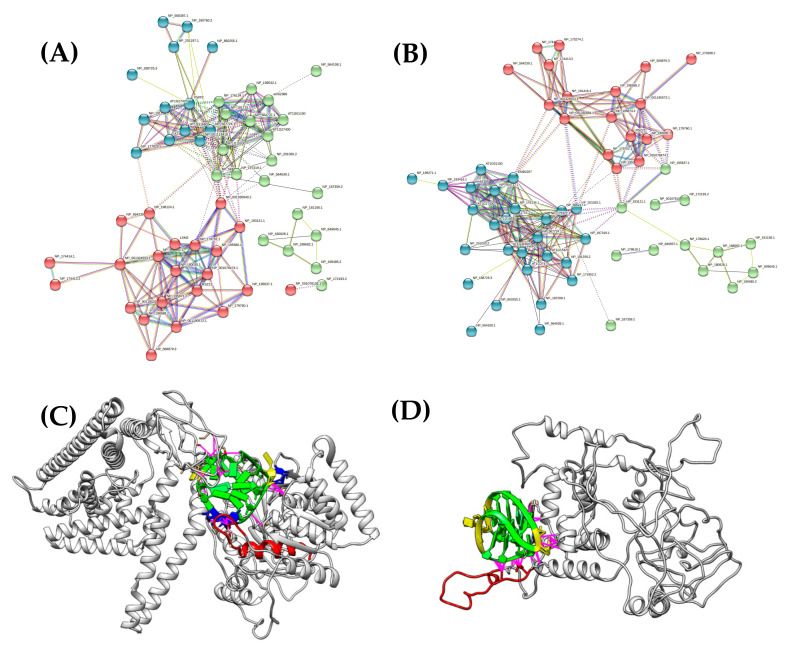
Interaction network of proteins containing G4-binding motifs. (**A**) Proteins containing NIQI (RGRGRGRGGGSGGSGGRGRG) motif. (**B**) Proteins containing RGG (RRGDGRRRGGGGRGQGGRGRGGGFKG) motif. (**C**) 3D model of HEN2 protein from *Arabidopsis thaliana* (OAP08511.1) with docked G4 RNA (NDB colors: guanines in green, cytosines in yellow, uracils in blue), predicted clashes/contacts are depicted by pink lines. G4-binding motif is depicted in the red (**D**) 3D model of KAKU4 protein from *Arabidopsis thaliana* (Q949W6.1) with docked G4 DNA (NDB colors: guanines in green and cytosine loops in yellow), predicted clashes/contacts are depicted by pink lines. G4-binding motif is depicted in red.

In general, based on the presence of a G4-binding motif, we identified more than 400 proteins with a theoretical potential to bind G4 structures in *Arabidopsis thaliana* (555 containing the significant RGG motif, and 408 containing the significant NIQI motif). The complete FIMO results can be found in the Supplementary Materials (Files S1 and S2). The functional protein association network (STRING) analysis revealed a high number of functionally interconnected proteins with specific molecular functions, such as DNA topology modifications, DNA/RNA binding, RNA metabolism, and ribosome biogenesis. The results of the STRING analyses are depicted in Figure 2A,B. The complete STRING results are listed in the Supplementary Materials (Files S3 and S4).

The STRING functional enrichment analysis, following the NIQI motif scanning, revealed 93 interconnected proteins with the potential to interact with G4s. These proteins can be divided into three groups: proteins able to bind/interact with DNA or RNA; proteins linked with ribosome biogenesis and maintenance; other proteins. The most interesting proteins from the first group (proteins able to interact with DNA or RNA) were the following: DNA topoisomerase 3α (involved in homologous recombination and torsion stretch relaxation [35]) and the plant-unique protein KAKU4 (responsible for nuclear morphology in plants [36]). In addition, our data also indicate the G4-interacting potential for the following: TF II D-subunit 15b (transcription initiation factor), RNA-binding family protein (RRM/RBD/RNP motifs) responsible for RNA splicing, the serine/arginine-rich splicing factor RSZ21 (mRNA processing [37]), the mediator of RNA pol II-transcription subunit 36a (mediator of RNA polymerase II), protein decapping 3α (translation repressor), and proteins belonging to the Argonaute family (AGO2 and AGO3, which contain the NIQI motif). In the second group of proteins (proteins linked with ribosome biogenesis and maintenance), the most interesting were: periodic tryptophan protein (processing of pre18S ribosomal RNA), ribosomal family proteins-60S acidic family protein, 60S ribosomal protein L17-1, 60S ribosomal protein L19-140S ribosomal protein S12-1, 40S ribosomal protein S2-1, and 40S ribosomal protein S2-3.

The STRING functional enrichment analysis, following the RGG motif scanning, revealed 96 proteins, which can be divided into three groups of proteins as described above. Some of them differed to the results from the NIQI motif search, while others were the same/similar. Additionally, they were involved in processes similar to those described in the paragraph above. Some of the promising G4-binding proteins identified were proteins GRP2 and GRP7. GRP2 is a glycine-rich protein that binds nucleic acids and promotes nucleic acid melting, and also binds to and unwinds RNA, ssDNA, and dsDNA [38]. GRP7 binds to RNAs and DNAs (preferentially to poly U or poly G), and is involved in alternative splicing [39]. Further interesting putative G4-binding proteins were: RSZp22 (RNA nucleocytoplasmic shuttling protein); RNA 2′-phosphotransferase (tRNA splicing); Argonaute family proteins, AGO1, AGO2, and AGO3 (small RNA binding [40]); NUC-L1 (rRNA processing, nucleolus organization [41]); RSZ22a (intron recognition and spliceosome assembly); EI4B1 (translation initiation).

To better understand the mechanistic basis of protein–G4 interactions, we carried out a molecular docking analysis of the two representative G4-binding proteins identified above. We selected RNA binding protein HEN2, which is an important ATP-dependent RNA helicase involved in the degradation of a large number of small non-coding RNAs (e.g., snoRNA and miRNA precursors), incompletely spliced mRNAs, and transcripts produced from pseudogenes and/or intergenic regions [42]. In plants, HEN2 regulates floral organ spacing and identity [43]. The docking of a parallel conformation of G4 RNA (GGGUCGGGUUGGGCGGG) to this protein showed that it suitably fits in an arginine-rich cavity (Figure 2C), and that the neighboring glycine residues may promote bending to allow the arginine residues to recognize G4. Second, we selected the protein, KAKU4, and docked it into the G4 DNA (GGGGCCGGGGCCGGGGCCGGGG). We found that, here, arginine residues also play an important role in the recognition of G4, particularly of Arg 222, Arg 238, Arg 279, and Arg 282 (see Figure 2D).

### 3.2. Identification of G4-Binding Proteins in Plants Using a BLASTp Approach

To validate the FIMO search results described above, we used another computational algorithm, BLASTp. The top 10 most statistically significant results (according to the E-value) for *Arabidopsis thaliana* are shown in Table 1, separately for the NIQI and RGG motif.

To predict the G4-binding proteins in non-model plant species, we performed an additional BLASTp search that was not limited to *Arabidopsis thaliana*, but applied to the whole *Viridiplantae* group (green plants); the results of which are shown in Table 2. Proteins with known function or process they are involved in comprised e.g. THO com-plex subunit 4a responsible for RNA binding (THO complex balances transcription and mRNA processing [44]). The next important example is the UVRD/REP-type putative helicase. Helicases of this type are responsible for DNA unwinding and require ATP to function [45]. Interestingly, UVRD is already known to be a G4-binding protein in *Escherichia coli* and *Neisseria gonorrhoeae*. The next important protein predicted to be G4-binding was Fibrillarin 2, which is involved in rRNA processing and has methyltransferase activity [46]. Further interesting examples of putative G4-binding proteins are: the mediator of RNA polymerase transcription subunit 36a-like, which is involved in rRNA methylation; HEN2 with RNA helicase activity [42]; a helicase-like transcription factor with DNA helicase activity; Keratin Type II cytoskeletal 1-like protein; translation initiation factor IF-2.

To further support our hypothesis regarding the occurrence of G4-binding proteins in plants sensu lato, we extended the BLASTp search to algae. The results are shown in Table 3. With the exception of hypothetical protein sequences, we found many known proteins with G4-binding potential. This included: NER endonuclease; Protein EXPORTIN 1A (XPO1); DNA helicase; HEN2 protein; helicase-like transcription factor; La1 protein homolog (required for embryogenesis in *Arabidopsis thaliana*, which binds to and protects the 3′ poly(U) terminus of nascent RNA polymerase III transcripts [47]); DEAD-box ATP-dependent RNA helicase 10; FAD-dependent oxidoreductase; the translation initiation factor. Our analysis also revealed that the G4-binding motif is present in the following interesting proteins that were not in the top 10, but are still significant: GRP2; GRP5; GRP7; GRP8; DNA topoisomerase IA; heat stress transcription factor B-2b (HSFB2B); RSZ22a (serine/arginine-rich splicing factor RSZ22A); glycine-rich RNA-binding protein 2, mitochondrial (GR-RBP2); THO complex subunit 4D (ALY4); DNA topoisomerase 3-alpha (TOP3A); protein decapping 5 (DCP5). The complete BLASTp searching results can be found in the Supplementary Materials (File S5–S10).

**Figure 3:**
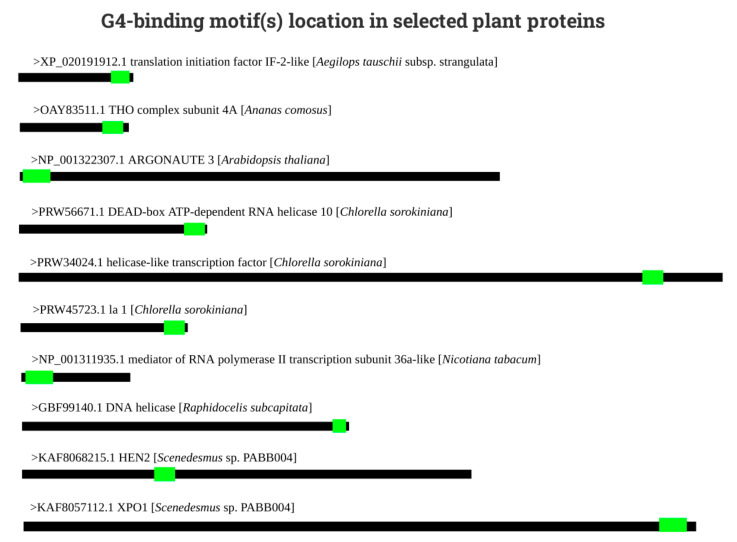
Location of the G4-binding motifs (light green boxes) in 10 randomly selected plant proteins (thick black lines). The shortest protein sequences (THO complex subunit 4a; translation initiation factor IF-2-like; mediator of RNA polymerase II transcription subunit 36a-like) are only approximately 300 aa residues long. The longest protein sequence (helicase-like transcription factor) is over 1700 aa residues long. Except for protein HEN2, all putative G4-binding motifs are located near N’ or C’ protein termini.

The analysis of the occurrence of the G4-binding motifs in 10 randomly selected plant proteins showed that these are present mainly in the N’ and C’ terminal regions (Figure 3). Particularly, in two proteins (ARGONAUTE 3 from *Arabidopsis thaliana*, and mediator of RNA polymerase II transcription subunit 36a-like from *Nicotiana tabacum*), the main G4-binding motif was predicted to be located in the N’ terminal regions. In seven proteins, the main G4-binding motif was located in their C’ terminal ends (e.g., protein la 1 from *Chlorella sorokiniana*), and only one protein contained a putative G4-binding motif in its central region. This is in correlation with our previous analysis dealing with the known human G4-binding proteins [23], although the evolutionarily and mechanistic explanation of this phenomenon is still lacking. One explanation could be the modular nature of virtually all proteins, suggesting that the arginine-glycine rich motifs were independently acquired during evolution to help organisms deal with G4s.

Lastly, we inspected the number of putative G4-binding motifs in green plants and algae (Supplementary Material File S11). According to this analysis, the highest number of G4-binding motifs was present in *Chara braunii*, the model organism for plant terrestrialization (196 significant hits in total), followed by *Zea mays* (149 hits), *Arabidopsis thaliana* (147 hits), and *Chlamydomonas reinhardtii* (143 hits). In the vast majority of plants, less than 30 G4-binding motifs were found. As some plant genomes were sequenced more in-depth, and due to other biases (quality of genome/proteome annotation), it is difficult to say if the differences observed in the total counts of G4-binding motifs have some physiological relevance.

## 4. Discussion

When searching for G-quadruplex binding proteins, we found many hypothetical proteins and proteins with unknown functions, but also some relatively well-characterized proteins. One well-characterized protein was the Alba DNA/RNA binding protein, which is involved in, for example, genome packaging and organization, and RNA metabolism [48]. Additional examples were Apoptosis inhibitory protein 5 (API5), which binds RNA/mRNA, and is involved in programmed cell death regulation [49]; and protein Argonaute 3 (AGO3), which binds nucleic acids, and provides RNA silencing and defense responses to viral infections [50]. There were also many proteins belonging to the glycine-arginine-rich family, such as GRP2, GRP5, GRP7, and GRP8. These multifunctional proteins are regulated by abiotic stresses [51,52], and are involved in the early development of *Arabidopsis thaliana* [53]. As these proteins are known to promote DNA melting, they could also bind and help to resolve/unfold plant G4s.

One of our predicted G4-binding proteins was KAKU4. It is known that KAKU4 plays a role in the modulation of nuclear morphology in *Arabidopsis thaliana*, and it is likely that KAKU4 acts, directly, as a component of the nuclear lamina-like structure in seed-bearing plants [36]. Our results suggest that KAKU4 could also bind G4 structures, and maintain the organization of nucleic acids via these interactions. In the future, it would be beneficial to inspect the G4-binding potential of KAKU4 in vitro (using standard molecular biology techniques as an electrophoretic mobility shift assay (EMSA), circular dichroism spectroscopy, and other approaches).

Interestingly, some of our predicted proteins (or their close homologs) are already known to bind G4s. DExH-box-dependent RNA helicase DExH1 from *Arabidopsis thaliana* share significant sequence homology with ATP-dependent DNA/RNA helicase DHX36 isoform 1 (also known as DHX36 or RHAU). There is also substantial evidence that human DHX36 binds/unwinds G4s via a non-processive, local strand-unwinding mechanism [54,55]. Another example is the nucleolin 1 protein (NUCL1). The human protein, nucleolin, is known to stabilize the G4 structures folded by the LTR promoter, and silence HIV-1 viral transcription [56]. In future studies, the evolution of glycine-arginine-rich domains/regions could be analyzed. For instance, one of our predicted G4-binding proteins was fibrillarin 2, and this protein is highly evolutionarily conserved. However, in the course of evolution, from archaea to eukaryotes, it acquired an additional N-terminal glycine and arginine-rich domain [44]. This would suggest that the fibrillarin homologs in the archaea lack G4-binding potential.

**Figure 4:**
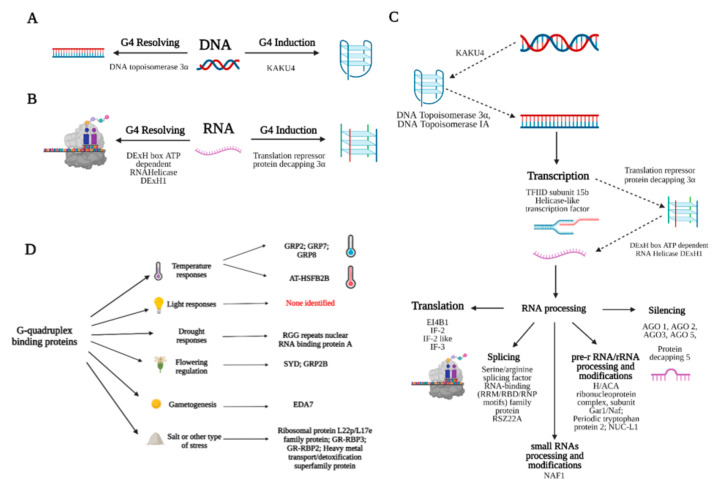
Proteins containing G4-binding motifs and their possible role in the induction and resolving of G4 nucleic acids in plants. (**A**) On the left, DNA G4s could be specifically recognized and resolved by DNA topoisomerases; on the right, G4s’ induction and stabilization could be promoted by KAKU4 protein. (**B**) RNA G4s could be resolved e.g., by the DExH box ATP-dependent RNA helicase DExH1. To facilitate RNA G4s’ forming and stabilization, translation repressor protein decapping 3α seems to be a good candidate. (**C**) Proteins containing G4 binding motif in the context of cellular processes. (**D**) Predicted G4-binding proteins are involved in many previously described biological functions, comprising temperature, drought, salt stress responses, flowering regulation, and gametogenesis.

G4s in DNA must be fine-tuned and balanced in the process of dynamic G4 stabilizing and resolving [57], ensuring the correct replication of DNA. Considering transcription, G4s also need to be specifically and spatiotemporally induced/resolved, allowing cells to utilize an additional regulatory network layer to regulate gene expression [58] (Figure 4A,B). In addition, it seems that many of the predicted G4-binding proteins participate in RNA processing pathways, including mRNA translation, splicing, small RNA processing and modification, pre-rRNA/rRNA processing and modifications, and in RNA silencing (Figure 4C).

Overall, we found G4-binding motifs in both green plants and algae. The majority of them have already described molecular functions, and we organized them into groups by their potential ability to form/induce G4 structures (KAKU4), resolve G4s (DNA topoisomerase 3α), or by their potential to drive biological processes (replication, transcription, RNA processing or translation), as depicted in Figure 4. It is, therefore, likely that G4–protein interactions are also of great physiological relevance in plants, as suggested in Figure 4D.

Based on our data, we hypothesize that G4–protein interactions play a significant role in molecular processes, such as apoptosis, RNA processing, silencing, and siRNA generation. On the other hand, these hypotheses should be confirmed directly by G4–protein interaction detection, such as using pull-down assays, as described in [59]. Nonetheless, such studies are limited to in vitro experiments, and the high-throughput detection of all G4–protein interactions in living cells is still nearly impossible.

## 5. Conclusions

We predicted more than 400 candidate proteins in green plants (including the model organism, *Arabidopsis thaliana*) and algae with the potential to interact with G-quadruplexes, using the combination of two in silico approaches (BLASTp and FIMO). Interestingly, these proteins form highly statistically significant functional networks, suggesting their involvement in key molecular and physiological processes, which was confirmed by their annotation. Some of the G4 interacting proteins are common for green plants and algae (for example, DNA/RNA-binding proteins), which strengthens our hypothesis regarding the shared G4-centric regulation that is ensured by G4-binding proteins. In addition, we have carried out some representative molecular docking to prove that these proteins are able to interact with G4s via their RGG-rich regions, and their conformational geometry enables such interaction. Further studies are needed to evaluate these predicted G4-binding proteins in vitro, followed by in vivo functional studies.

## Figures and Tables

**Table 1:** Predicted G4-binding proteins in *Arabidopsis thaliana*. Top 10 NIQI and RGG motif-containing proteins in *Arabidopsis thaliana* BLASTp searching results.

Motif	Protein Full Name	AN	Length	E-Value	Query Cover
**NIQI**	Hypothetical protein AXX17 AT1G21190	OAP13840.1	265	4 × 10^−6^	100
	Unnamed protein product	AAN60302.1	272	4 × 10^−6^	100
	Unnamed protein product	CAA0224497.1	312	4 × 10^−6^	100
	Alba DNA/RNA binding protein	NP_564108.1	315	4 × 10^−^^6^	100
	Unnamed protein product	VYS46658.1	317	4 × 10^−^^6^	100
	Contains similarity to pigpen protein from *M. musculus*	AAF79893.1	538	4 × 10^−^^6^	100
	Unnamed protein product	CAD5313245.1	787	4 × 10^−^^6^	100
	F1K23.1	AAF24538.2	347	1 × 10^−^^5^	100
	Hypothetical protein	AAF88129.1	533	1 × 10^−^^5^	100
	Apoptosis inhibitory protein 5	NP_001322605.1	534	1 × 10^−^^5^	100
**RGG**	Argonaute 3	NP_001322307.1	1158	2 × 10^−6^	84
	Unnamed protein product	VYS47693.1	1205	5 × 10^−6^	84
	Argonaute 3	NP_174414.1	1194	1 × 10^−5^	84
	Unnamed protein product	CAA0259614.1	1194	1 × 10^−5^	84
	Argonaute 3	NP_001322306.1	1120	3 × 10^−5^	84
	AGO3	OAP18010.1	1141	4 × 10^−5^	84
	Glycine-rich protein	NP_568523.1	118	4 × 10^−4^	84
	Unnamed protein product	VYS68274.1	118	4 × 10^−4^	84
	Hypothetical protein AXX17 AT5G32160	OAO90547.1	123	4 × 10^−^^4^	84
	Hyaluronan/mRNA binding family	NP_001078399.1	265	9 × 10^−^^4^	80

**Table 2:** Comparison of NIQI and RGG motifs containing proteins in green plants BLASTp searching.

Motif	Protein Name	Organism	Simple Name	AN	Length	E-Value	Query Cover
**NIQI**	Uncharacterized protein	*Cynara cardunculus*	Globe artichoke	XP_024965254.1	265	2 × 10^−7^	100
	Uncharacterized protein	*Asparagus officinalis*	Garden asparagus	ONK65585.1	310	2 × 10^−7^	100
	Keratin, type II cytoskeletal 1-like	*Asparagus officinalis*	Garden asparagus	XP_020272794.1	301	2 × 10^−7^	100
	Hypothetical protein	*Chlamydomonas incerta*	-	KAG2446147.1	2307	5 × 10^−7^	100
	THO complex subunit 4a	*Ananas comosus*	Pineapple	OAY83511.1	271	5 × 10^−7^	100
	Translation initiation factor IF-2-like	*Aegilops tauschii*	Tausch’s goatgrass	XP_020191912.1	278	2 × 10^−6^	100
	Unnamed protein product	*Lactuca saligna*	Willowleaf lettuce	CAB4097197.1	728	3 × 10^−6^	100
	Hypothetical protein	*Chlamydomonas schloesseri*	-	KAG2434571.1	2954	4 × 10^−6^	100
	UVRD/REP type putative DNA helicase	*Klebsormidium nitens*	-	GAQ80746.1	1395	4 × 10^−6^	100
	Hypothetical protein	*Edaphochlamys debaryana*	-	KAG2484473.1	1151	4 × 10^−6^	100
**RGG**	Hypothetical protein	*Pycnococcus provasolii*	-	GHP06452.1	396	4 × 10^−8^	88
	Unnamed protein product	*Triticum aestivum*	Common wheat	CDM81338.1	300	6 × 10^−6^	84
	Unnamed protein product	*Microthlaspi erraticum*	-	CAA7061275.1	1044	9 × 10^−6^	84
	Fibrillarin 2	*Nicotiana benthamiana*	Benth	CAR92137.1	314	2 × 10^−5^	96
	Mediator of RNA polymerase II transcription subunit 36a-like	*Nicotiana tabacum*	Cultivated tobacco	NP_001311935.1	314	2 × 10^−5^	96
	Predicted mediator of RNA polymerase II transcription subunit 36a-like	*Nicotiana attenuata*	Coyote tobacco	XP_019243309.1	314	2 × 10^−5^	96
	Mediator of RNA polymerase II transcription subunit 36a-like	*Nicotiana tomentosiformis*	-	XP_009625367.1	314	2 × 10^−5^	96
	Fibrillarin 2	*Nicotiana benthamiana*	Benth	CAK32531.1	314	2 × 10^−5^	96
	HEN2	*Scenedesmus* sp.	-	KAF8068215.1	1082	2 × 10^−5^	84
	Helicase-like transcription factor	*Chlorella sorokiniana*	-	PRW34024.1	1709	3 × 10^−5^	88

**Table 3:** Comparison of NIQI and RGR motifs containing proteins in algae BLASTp searching.

Motif	Protein Name	Organism	AN	Length	E-Value	Query Cover
**NIQI**	Hypothetical protein	*Chlamydomonas incerta*	KAG2446147.1	2307	2 × 10^−8^	100
	Hypothetical protein	*Chlamydomonas schloesseri*	KAG2434571.1	2954	2 × 10^−7^	100
	Hypothetical protein	*Edaphochlamys debaryana*	KAG2484473.1	1151	2 × 10^−7^	100
	NER endonuclease	*Chlamydomonas reinhardtii*	ALL55223.1	2463	3 × 10^−7^	100
	Hypothetical protein	*Chlamydomonas schloesseri*	KAG2440529.1	2398	6 × 10^−7^	100
	XPO1	*Scenedesmus* sp.	KAF8057112.1	1615	9 × 10^−7^	100
	DNA helicase	*Raphidocelis subcapitata*	GBF99140.1	787	9 × 10^−7^	100
	Hypothetical protein	*Edaphochlamys debaryana*	KAG2500053.1	1082	1 × 10^−6^	100
	Hypothetical protein	*Edaphochlamys debaryana*	KAG2486618.1	1053	1 × 10^−6^	100
	Hypothetical protein	*Chlamydomonas incerta*	KAG2433306.1	1398	2 × 10^−6^	100
**RGG**	Hypothetical protein	*Pycnococcus provasolii*	GHP06452.1	396	2 × 10^−9^	88
	HEN2	*Scenedesmus* sp.	KAF8068215.1	1082	8 × 10^−7^	84
	Helicase-like transcription factor	*Chlorella sorokiniana*	PRW34024.1	1709	1 × 10^−6^	88
	Hypothetical protein	*Chlamydomonas incerta*	KAG2443577.1	671	3 × 10^−6^	84
	Hypothetical protein	*Chlamydomonas schloesseri*	KAG2451206.1	782	4 × 10^−6^	88
	La1	*Chlorella sorokiniana*	PRW45723.1	398	1 × 10^−5^	92
	DEAD-box ATP-dependent RNA helicase 10	*Chlorella sorokiniana*	PRW56671.1	462	2 × 10^−5^	88
	Hypothetical protein	*Edaphochlamys debaryana*	KAG2497970.1	830	2 × 10^−5^	96
	FAD dependent oxidoreductase	*Raphidocelis subcapitata*	GBF89971.1	859	2 × 10^−5^	84
	Translation initiation factor 3	*Ostreococcus tauri*	XP_003078378.1	266	3 × 10^−5^	80

## Data Availability

Detailed information can be provided by corresponding author.

## Supplementary Materials

The following are available online at https://www.mdpi.com/article/10.3390/biotech10040020/s1. File S1: Table containing FIMO results (NIQI motif scanning); File S2: Table containing FIMO results (RGG motif scanning); File S3: File containing annotations from STRING (NIQI motif); File S4: File containing annotations from STRING (RGG motif); File S5: File with complete BLASTp search results shown in Table 1 (proteins containing NIQI motif); File S6: File with complete BLASTp search results shown in Table 1 (proteins containing RGG motif); File S7: File with complete BLASTp search results shown in Table 2 (proteins containing NIQI motif); File S8: File with complete BLASTp search results shown in Table 2 (proteins containing RGG motif); File S9: File with complete BLASTp search results shown in Table 3 (proteins containing NIQI motif); File S10: File with complete BLASTp search results shown in Table 3 (proteins containing RGG motif); File S11: Total counts of G4-binding motifs found in particular organisms.

## References

[1] Rhodes D., Lipps H.J. (2015). G-Quadruplexes and Their Regulatory Roles in Biology. Nucleic Acids Res..

[2] Fujii T., Podbevšek P., Plavec J., Sugimoto N. (2017). Effects of Metal Ions and Cosolutes on G-Quadruplex Topology. J. Inorg. Biochem..

[3] Bartas M., Brázda V., Karlický V., Červeň J., Pečinka P. (2018). Bioinformatics Analyses and In Vitro Evidence for Five and Six Stacked G-Quadruplex Forming Sequences. Biochimie.

[4] Guedin A., Gros J., Alberti P., Mergny J.-L. (2010). How Long Is Too Long? Effects of Loop Size on G-Quadruplex Stability. Nucleic Acids Res..

[5] Burge S., Parkinson G.N., Hazel P., Todd A.K., Neidle S. (2006). Quadruplex DNA: Sequence, Topology and Structure. Nucleic Acids Res..

[6] Patro L.P.P., Kumar A., Kolimi N., Rathinavelan T. (2017). 3D-NuS: A Web Server for Automated Modeling and Visualization of Non-Canonical 3-Dimensional Nucleic Acid Structures. J. Mol. Biol..

[7] Hänsel-Hertsch R., Simeone A., Shea A., Hui W.W., Zyner K.G., Marsico G., Rueda O.M., Bruna A., Martin A., Zhang X. (2020). Landscape of G-Quadruplex DNA Structural Regions in Breast Cancer. Nat. Genet..

[8] Simone R., Fratta P., Neidle S., Parkinson G.N., Isaacs A.M. (2015). G-Quadruplexes: Emerging Roles in Neurodegenerative Diseases and the Non-Coding Transcriptome. FEBS Lett..

[9] Wang E., Thombre R., Shah Y., Latanich R., Wang J. (2021). G-Quadruplexes as Pathogenic Drivers in Neurodegenerative Disorders. Nucleic Acids Res..

[10] Lavezzo E., Berselli M., Frasson I., Perrone R., Palù G., Brazzale A.R., Richter S.N., Toppo S. (2018). G-Quadruplex Forming Sequences in the Genome of All Known Human Viruses: A Comprehensive Guide. PLoS Comput. Biol..

[11] Bartas M., Brázda V., Bohálová N., Cantara A., Volná A., Stachurová T., Malachová K., Jagelská E.B., Porubiaková O., Červeň J. (2020). In-Depth Bioinformatic Analyses of Nidovirales Including Human SARS-CoV-2, SARS-CoV, MERS-CoV Viruses Suggest Important Roles of Non-Canonical Nucleic Acid Structures in Their Lifecycles. Front. Microbiol..

[12] Brázda V., Hároníková L., Liao J.C., Fojta M. (2014). DNA and RNA Quadruplex-Binding Proteins. Int. J. Mol. Sci..

[13] Mishra S.K., Tawani A., Mishra A., Kumar A. (2016). G4IPDB: A Database for G-Quadruplex Structure Forming Nucleic Acid Interacting Proteins. Sci. Rep..

[14] Cagirici H.B., Budak H., Sen T.Z. (2021). Genome-Wide Discovery of G-Quadruplexes in Barley. Sci. Rep..

[15] Cagirici H.B., Sen T.Z. (2020). Genome-Wide Discovery of G-Quadruplexes in Wheat: Distribution and Putative Functional Roles. G3 Genes Genomes Genet..

[16] Stefos G.C., Theodorou G., Politis I. (2021). DNA G-Quadruplexes: Functional Significance in Plant and Farm Animal Science. Anim. Biotechnol..

[17] Volná A., Bartas M., Karlický V., Nezval J., Kundrátová K., Pečinka P., Špunda V., Červeň J. (2021). G-Quadruplex in Gene Encoding Large Subunit of Plant RNA Polymerase II: A Billion-Year-Old Story. Int. J. Mol. Sci..

[18] Zhang Y., Yang M., Duncan S., Yang X., Abdelhamid M.A.S., Huang L., Zhang H., Benfey P.N., Waller Z.A.E., Ding Y. (2019). G-Quadruplex Structures Trigger RNA Phase Separation. Nucleic Acids Res..

[19] Andorf C.M., Kopylov M., Dobbs D., Koch K.E., Stroupe M.E., Lawrence C.J., Bass H.W. (2014). G-Quadruplex (G4) Motifs in the Maize (*Zea mays* L.) Genome Are Enriched at Specific Locations in Thousands of Genes Coupled to Energy Status, Hypoxia, Low Sugar, and Nutrient Deprivation. J. Genet. Genom..

[20] Cho H., Cho H.S., Nam H., Jo H., Yoon J., Park C., Dang T.V.T., Kim E., Jeong J., Park S. (2018). Translational Control of Phloem Development by RNA G-Quadruplex–JULGI Determines Plant Sink Strength. Nat. Plants.

[21] Sjakste T., Leonova E., Petrovs R., Trapina I., Röder M.S., Sjakste N. (2020). Tight DNA-Protein Complexes Isolated from Barley Seedlings Are Rich in Potential Guanine Quadruplex Sequences. PeerJ.

[22] Vasilyev N., Polonskaia A., Darnell J.C., Darnell R.B., Patel D.J., Serganov A. (2015). Crystal Structure Reveals Specific Recognition of a G-Quadruplex RNA by a β-Turn in the RGG Motif of FMRP. Proc. Natl. Acad. Sci. USA.

[23] Brázda V., Červeň J., Bartas M., Mikysková N., Coufal J., Pečinka P. (2018). The Amino Acid Composition of Quadruplex Binding Proteins Reveals a Shared Motif and Predicts New Potential Quadruplex Interactors. Molecules.

[24] Grant C.E., Bailey T.L., Noble W.S. (2011). FIMO: Scanning for Occurrences of a given Motif. Bioinformatics.

[25] Bailey T.L., Johnson J., Grant C.E., Noble W.S. (2015). The MEME Suite. Nucleic Acids Res..

[26] O’Leary N.A., Wright M.W., Brister J.R., Ciufo S., Haddad D., McVeigh R., Rajput B., Robbertse B., Smith-White B., Ako-Adjei D. (2016). Reference Sequence (RefSeq) Database at NCBI: Current Status, Taxonomic Expansion, and Functional Annotation. Nucleic Acids Res..

[27] Johnson M., Zaretskaya I., Raytselis Y., Merezhuk Y., McGinnis S., Madden T.L. (2008). NCBI BLAST: A Better Web Interface. Nucleic Acids Res..

[28] Zhang Y., Gaetano C.M., Williams K.R., Bassell G.J., Mihailescu M.R. (2014). FMRP Interacts with G-Quadruplex Structures in the 3′-UTR of Its Dendritic Target Shank1 MRNA. RNA Biol..

[29] Szklarczyk D., Franceschini A., Wyder S., Forslund K., Heller D., Huerta-Cepas J., Simonovic M., Roth A., Santos A., Tsafou K.P. (2015). STRING V10: Protein–Protein Interaction Networks, Integrated over the Tree of Life. Nucleic Acids Res..

[30] Källberg M., Wang H., Wang S., Peng J., Wang Z., Lu H., Xu J. (2012). Template-Based Protein Structure Modeling Using the RaptorX Web Server. Nat. Protoc..

[31] Lambert C., Leonard N., De Bolle X., Depiereux E. (2002). ESyPred3D: Prediction of Proteins 3D Structures. Bioinformatics.

[32] Yan Y., Tao H., He J., Huang S.-Y. (2020). The HDOCK Server for Integrated Protein–Protein Docking. Nat. Protoc..

[33] Mullen M.A., Olson K.J., Dallaire P., Major F., Assmann S.M., Bevilacqua P.C. (2010). RNA G-Quadruplexes in the Model Plant Species *Arabidopsis thaliana*: Prevalence and Possible Functional Roles. Nucleic Acids Res..

[34] Pettersen E.F., Goddard T.D., Huang C.C., Couch G.S., Greenblatt D.M., Meng E.C., Ferrin T.E. (2004). UCSF Chimera—a Visualization System for Exploratory Research and Analysis. J. Comput. Chem..

[35] Costa-Silva H.M., Resende B.C., Umaki A.C.S., Prado W., da Silva M.S., Virgílio S., Macedo A.M., Pena S.D.J., Tahara E.B., Tosi L.R.O. (2021). DNA Topoisomerase 3α Is Involved in Homologous Recombination Repair and Replication Stress Response in Trypanosoma Cruzi. Front. Cell Dev. Biol..

[36] Goto C., Tamura K., Fukao Y., Shimada T., Hara-Nishimura I. (2014). The Novel Nuclear Envelope Protein KAKU4 Modulates Nuclear Morphology in Arabidopsis. Plant Cell.

[37] Long J.C., Caceres J.F. (2009). The SR Protein Family of Splicing Factors: Master Regulators of Gene Expression. Biochem. J..

[38] Sasaki K., Liu Y., Kim M.-H., Imai R. (2015). An RNA Chaperone, AtCSP2, Negatively Regulates Salt Stress Tolerance. Plant Signal. Behav..

[39] Köster T., Meyer K., Weinholdt C., Smith L.M., Lummer M., Speth C., Grosse I., Weigel D., Staiger D. (2014). Regulation of Pri-MiRNA Processing by the HnRNP-like Protein AtGRP7 in Arabidopsis. Nucleic Acids Res..

[40] Cao J.-Y., Xu Y.-P., Cai X.-Z. (2020). Integrated MiRNAome and Transcriptome Analysis Reveals Argonaute 2-Mediated Defense Responses Against the Devastating Phytopathogen Sclerotinia Sclerotiorum. Front. Plant Sci..

[41] Pontvianne F., Matía I., Douet J., Tourmente S., Medina F.J., Echeverria M., Sáez-Vásquez J. (2007). Characterization of AtNUC-L1 Reveals a Central Role of Nucleolin in Nucleolus Organization and Silencing of AtNUC-L2 Gene in Arabidopsis. Mol. Biol. Cell.

[42] Lange H., Zuber H., Sement F.M., Chicher J., Kuhn L., Hammann P., Brunaud V., Bérard C., Bouteiller N., Balzergue S. (2014). The RNA Helicases AtMTR4 and HEN2 Target Specific Subsets of Nuclear Transcripts for Degradation by the Nuclear Exosome in *Arabidopsis thaliana*. PLoS Genet..

[43] Western T.L., Cheng Y., Liu J., Chen X. (2002). HUA ENHANCER2, a Putative DExH-Box RNA Helicase, Maintains Homeotic B and C Gene Expression in Arabidopsis. Development.

[44] Larochelle M., Lemay J.-F., Bachand F. (2012). The THO Complex Cooperates with the Nuclear RNA Surveillance Machinery to Control Small Nucleolar RNA Expression. Nucleic Acids Res..

[45] Shukla K., Thakur R.S., Ganguli D., Rao D.N., Nagaraju G. (2017). *Escherichia coli* and *Neisseria gonorrhoeae* UvrD Helicase Unwinds G4 DNA Structures. Biochem. J..

[46] Shubina M.Y., Musinova Y.R., Sheval E.V. (2016). Nucleolar Methyltransferase Fibrillarin: Evolution of Structure and Functions. Biochem. Mosc..

[47] Fleurdépine S., Deragon J.-M., Devic M., Guilleminot J., Bousquet-Antonelli C. (2007). A Bona Fide La Protein Is Required for Embryogenesis in *Arabidopsis thaliana*. Nucleic Acids Res..

[48] Goyal M., Banerjee C., Nag S., Bandyopadhyay U. (2016). The Alba Protein Family: Structure and Function. Biochim. Biophys. Acta (BBA)—Prot. Proteom..

[49] Li X., Gao X., Wei Y., Deng L., Ouyang Y., Chen G., Li X., Zhang Q., Wu C. (2011). Rice APOPTOSIS INHIBITOR5 Coupled with Two DEAD-Box Adenosine 5′-Triphosphate-Dependent RNA Helicases Regulates Tapetum Degeneration. Plant Cell.

[50] Garcia-Ruiz H., Carbonell A., Hoyer J.S., Fahlgren N., Gilbert K.B., Takeda A., Giampetruzzi A., Ruiz M.T.G., McGinn M.G., Lowery N. (2015). Roles and Programming of Arabidopsis ARGONAUTE Proteins during Turnip Mosaic Virus Infection. PLoS Pathog..

[51] Kim J.S., Jung H.J., Lee H.J., Kim K.A., Goh C.-H., Woo Y., Oh S.H., Han Y.S., Kang H. (2008). Glycine-Rich RNA-Binding Protein7 Affects Abiotic Stress Responses by Regulating Stomata Opening and Closing in *Arabidopsis thaliana*. Plant J..

[52] Kim J.Y., Park S.J., Jang B., Jung C.-H., Ahn S.J., Goh C.-H., Cho K., Han O., Kang H. (2007). Functional Characterization of a Glycine-Rich RNA-Binding Protein 2 in *Arabidopsis thaliana* under Abiotic Stress Conditions. Plant J..

[53] Ciuzan O., Ladomery M., Wilson I., Hancock J., Pamfil D. (2013). The *Arabidopsis thaliana* Glycine-Rich RNA Binding Proteins AtGRP7 and AtGRP2 Are Involved in Early Development. ProEnviron. Promediu.

[54] Chen M.C., Murat P., Abecassis K., Ferré-D’Amaré A.R., Balasubramanian S. (2015). Insights into the Mechanism of a G-Quadruplex-Unwinding DEAH-Box Helicase. Nucleic Acids Res..

[55] Chen M.C., Tippana R., Demeshkina N.A., Murat P., Balasubramanian S., Myong S., Ferré-D’Amaré A.R. (2018). Structural Basis of G-Quadruplex Unfolding by the DEAH/RHA Helicase DHX36. Nature.

[56] Tosoni E., Frasson I., Scalabrin M., Perrone R., Butovskaya E., Nadai M., Palù G., Fabris D., Richter S.N. (2015). Nucleolin Stabilizes G-Quadruplex Structures Folded by the LTR Promoter and Silences HIV-1 Viral Transcription. Nucleic Acids Res..

[57] Di Antonio M., Ponjavic A., Radzevičius A., Ranasinghe R.T., Catalano M., Zhang X., Shen J., Needham L.-M., Lee S.F., Klenerman D. (2020). Single-Molecule Visualization of DNA G-Quadruplex Formation in Live Cells. Nat. Chem..

[58] Ravichandran S., Ahn J.-H., Kim K.K. (2019). Unraveling the Regulatory G-Quadruplex Puzzle: Lessons from Genome and Transcriptome-Wide Studies. Front. Genet..

[59] Serikawa T., Spanos C., von Hacht A., Budisa N., Rappsilber J., Kurreck J. (2018). Comprehensive Identification of Proteins Binding to RNA G-Quadruplex Motifs in the 5′ UTR of Tumor-Associated MRNAs. Biochimie.

